# Thromboelastometric analysis of the risk factors for return of spontaneous circulation in adult patients with out-of-hospital cardiac arrest

**DOI:** 10.1371/journal.pone.0175257

**Published:** 2017-04-05

**Authors:** Hiroyuki Koami, Yuichiro Sakamoto, Ryota Sakurai, Miho Ohta, Hisashi Imahase, Mayuko Yahata, Mitsuru Umeka, Toru Miike, Futoshi Nagashima, Takashi Iwamura, Kosuke Chris Yamada, Satoshi Inoue

**Affiliations:** 1 Department of Emergency and Critical Care Medicine, Faculty of Medicine, Saga University, Saga, Japan; 2 Advanced Emergency Care Center, Saga University Hospital, Saga, Japan; 3 Division of Trauma Surgery and Surgical Critical Care, Faculty of Medicine, Saga University, Saga, Japan; Azienda Ospedaliero Universitaria Careggi, ITALY

## Abstract

It is well known that coagulopathy is observed in patients with out-of-hospital cardiac arrest (OHCA). Thrombolytic therapy for those patients has been controversial until now. The purpose of this study was to identify a significant predictor for return of spontaneous circulation (ROSC) of OHCA patients in the emergency department (ED) using whole blood viscoelastic testing. Adult non-trauma OHCA patients transported to our hospital that underwent thromboelastometry (ROTEM) during cardiopulmonary resuscitation between January 2013 and December 2015 were enrolled in this study. We divided patients into two groups based on the presence or absence of ROSC, and performed statistical analysis utilizing patient characteristics, prehospital data, laboratory data, and ROTEM data. Seventy-five patients were enrolled. The ROSC group and non-ROSC group included 23 and 52 patients, respectively. The logistic regression analysis, utilizing significant parameters by univariate analysis, demonstrated that lactate level [odds ratio (OR) 0.880, 95% confidence interval (CI) 0.785–0.986, p = 0.028] and A30 of EXTEM test [OR 1.039, 95% CI 1.010–1.070, p = 0.009] were independent risk factors for ROSC. The cut-off values of lactate and A30 in EXTEM were 12.0 mmol/L and A 48.0 mm, respectively. We defined a positive prediction for ROSC if the patient presented lower lactate level (<12.0 mmol/L) and higher A30 of EXTEM (≥48.0 mm) with high specificity (94.7%) and accuracy (75.0%). The present study showed that lactate level and ROTEM parameter of clot firmness were reliable predictors of ROSC in the ED for adult patients with OHCA.

## Introduction

Out-of-hospital cardiac arrest (OHCA), which is due to multiple causes, presents dynamic pathological aspects in every organ system during their clinical course [1–3]. Interpretation of an individual’s pathophysiological conditions is challenging for emergency physician, as well as other medical personnel. It is well known that fibrin clot formation induced by activated extrinsic coagulation pathway and/or hyperfibrinolysis with systemic hypoperfusion are observed in patients with OHCA [3, 4]. However, antithrombotic treatment for patient with OHCA has been controversial [5–12].

Rotational thromboelastometry (ROTEM^®^; TEM International, GmbH, Munich, Germany) is a viscoelastic testing device utilizing citrated whole blood samples. There have been more than one thousand articles about this point-of-care test in various fields of medicine [13–16]. The features of this device can contribute to prompt pathophysiological evaluations of coagulation and fibrinolytic status compared with the conventional plasma based coagulation test [17, 18]. However, there is little evidence about coagulopathy of OHCA patients using viscoelastic devices such as ROTEM [19]. Moreover, no evidence was found for the relationship between the presence of coagulopathy (hypercoagulable? or hyperfibrinolytic?) and a possibility of return of spontaneous circulation (ROSC) until now.

The main goal of this study is to perform univariate and multivariate analyses in order to identify significant predictive indicators for ROSC utilizing patient characteristics, variables in prehospital care, standard laboratory tests, and ROTEM findings.

## Materials and methods

### Patients and study setting

This retrospective study has been certified by the institutional review board of Saga University (20140907). The study population included out-of-hospital cardiac arrest (OHCA) patients who were transported to the emergency department (ED) of Saga University Hospital between January 2013 and December 2015. Blood samples were obtained from patients on admission in order to perform a complete blood count, standard coagulation tests, blood gas, and ROTEM analysis during resuscitation. The indication for ROTEM test was determined by every physician in charge. We retrospectively evaluated the demographics, past medical history, data on prehospital care, and the laboratory tests including ROTEM findings. Exclusion groups included trauma, pregnancy, and patients under 18 years old.

### Japanese prehospital care in a case of out-of-hospital cardiac arrest

In general, prehospital resuscitation based on basic life support (BLS) tends to be performed by emergency medical services (EMS) on OHCA patients. Sometimes EMS activates the Dr. Car system, which is a prehospital patient care system in Japan that utilizes an ambulance in which an emergency physician and nurse, as well as paramedics, are on board. If Dr. Car is activated, medical personnel perform cardiopulmonary resuscitation according to the advanced cardiac life support (ACLS) at the scene. Our hospital launched this system in 2011 and we perform 300 to 350 missions a year since 2011 to the present.

### Variables in prehospital care by Japanese emergency medical service

Data on prehospital care performed by Japanese EMS were obtained. The following parameters were used: the presence of bystander CPR (expressed as numbers and percentages), the presence of Dr. Car activation (expressed as numbers and percentages), initial rhythm at scene and ED (categorized as ventricular fibrillation (VF)/ ventricular tachycardia (VT), pulseless electric activity (PEA) and asystole; expressed as numbers and percentages) and time from onset to blood sampling after ED admission (categorized as 30 minutes or less, from 31 to 60 minutes, from 61 to 120 minutes, 121 minutes or more; expressed as numbers and percentages). The definition of return of spontaneous circulation (ROSC) includes the presence of pulse of carotid artery and measurable blood pressure by an automated digital manometer at upper limb.

### Standard blood tests

Blood tests included white blood cell (WBC, expressed as /μL, normal range: 3300-8600/μL), hemoglobin (Hb, expressed as g/dL, normal range: 13.7–16.8 g/dL), platelet count (expressed as 10^4^/μL, normal range: 13.1–36.2x10^4^/μL), prothrombin time-international normalized ratio (PT-INR, normal range: 0.90–1.10), activated partial thromboplastin time (APTT, expressed as second (s), normal range: 25.0–40.0 s), fibrinogen (expressed as mg/dL, normal range: 200–400 mg/dL), fibrin degradation products (FDP, expressed as μg/mL, normal range: 0.0–5.0 μg/mL), D-dimer (DD, expressed as μg/mL, normal range: 0.00–1.00 μg/mL) and lactate (expressed as mmol/L, normal range: 0.5–1.6 mmol/L).

WBC, Hb and platelet were measured by the Sysmex XN-9000 (SYSMEX CORPORATION, Kobe, Japan); values of PT-INR, APTT, FDP and DD were obtained by the CP3000 (SEKISUI MEDICAL CO., LTD., Tokyo, Japan); and lactate level was measured by the ABL825 (Radiometer CO., LTD., Tokyo, Japan). The following reagents were used for coagulation tests: PT-INR (Thromborel^®^ S, Siemens Healthcare Diagnostics Products GmbH, Marburg, Germany), APTT (Coagpia^®^ APTT-N, SEKISUI MEDICAL CO., LTD., Tokyo, Japan), fibrinogen (Coagpia^®^ Fbg, SEKISUI MEDICAL CO., LTD., Tokyo, Japan), FDP (Nanopia^®^ P-FDP, SEKISUI MEDICAL CO., LTD., Tokyo, Japan) and DD (Nanopia^®^ D-dimer, SEKISUI MEDICAL CO., LTD., Tokyo, Japan).

### Thromboelastometry (ROTEM) analysis

Thromboelastometric evaluation was focused on the extrinsic coagulation pathway (EXTEM) and function of fibrinogen pathway in the extrinsic pathway (FIBTEM). Hyperfibrinolysis was defined as below 45 mm of amplitude (A) 10 in EXTEM test, over 600 seconds of clotting time (CT) in FIBTEM test, or more than 15% of maximum lysis (ML) within 60 minutes.[20] All tests were initiated less than one hour after admission and ran more than 60 minutes at 37°C.

### Statistical assessment

All continuous variables between both groups are represented as median (interquartile range (IQR)) and categorical variables as number (percentages). The P values were calculated from the Mann-Whitney U test for continuous variables, and Fisher’s exact test and Chi-square tests were used for categorical variables. Values of P<0.05 were considered to be significant. The factors with significant differences in the univariate analysis were analyzed in a multivariate analysis. We selected the logistic regression analysis (Forward: LR method) for multivariate analysis. Cut-off values were calculated using the factors with significant differences in the multivariate analysis. IBM SPSS Statistics version 22 (IBM Corp., Armonk, NY, USA) was utilized for statistical analyses.

## Results

**Fig 1:**
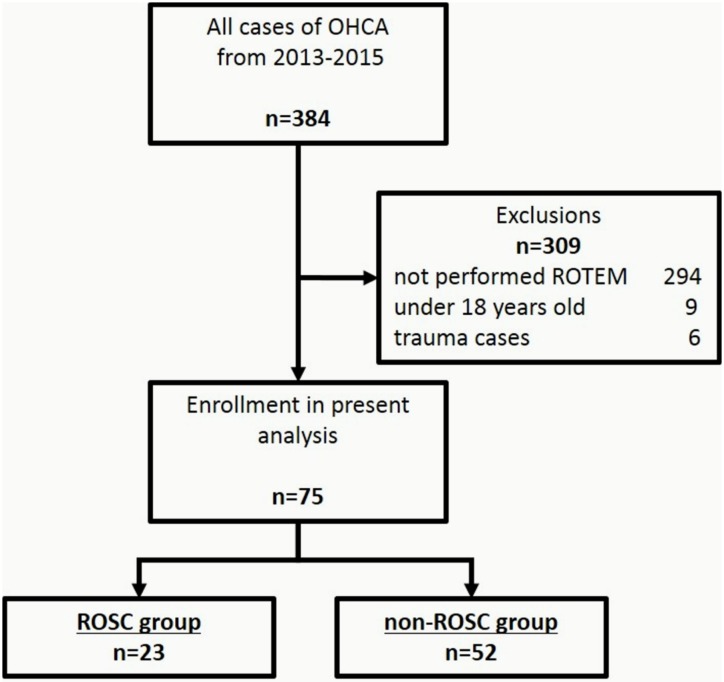
Study design. Seventy-five adult OHCA patients were analyze in this study. OHCA: out-of-hospital cardiac arrest; ROTEM: rotational thromboelastometry; ROSC: return of spontaneous circulation.

A total of 384 adult patients with OHCA were transported to the ED during the study observation period (Fig 1). Out of them, 309 cases (80.5%) met the exclusion criteria. Finally, 75 patients with ROTEM performed during the cardio pulmonary resuscitation in the ED were enrolled in this study. Out of all patients, ROSC was observed in 23 patients (30.7%). Next, included patients were divided into two groups based on the accomplishment of ROSC in the ED; ROSC group (n = 23) and non-ROSC group (n = 52).

### Patient characteristics and prehospital care between ROSC and non-ROSC groups

Table 1 shows results of univariate analysis on patient characteristics and prehospital care by EMS between ROSC and non-ROSC groups. ROSC after admission to the ED was not significantly related to age, gender, medication history, or presence of liver cirrhosis. Bystander CPR was performed on more than half of the patients within each group, however there was no statistical significance (ROSC; 59.1% vs. non-ROSC; 51.0%). In addition, there were no significant differences in the percentages of Dr. Car activation in both groups (21.7% vs. 21.2%, respectively). Although there tended to be more patients in non-ROSC group with asystole (84.0%) as an initial monitor rhythm at scene as compared with ROSC group (60.9%), no significant difference was observed. The same tendency was confirmed for initial rhythm in the ED. About 65% of the patients in ROSC group were transported to our ED within 60 minutes compared with non-ROSC group (36.5%), but this was also not significant.

**Table 1:** Patient characteristics and records of prehospital care between the ROSC and non-ROSC groups.

Variable	ROSC n = 23	non-ROSC n = 52	P
Age, y, median (IQR)	78 (64–85)	79 (61–86)	0.890
Male gender, n (%)	12 (52.2)	39 (75.0)	0.051
Medication, n (%)			
Aspirin/ Clopidogrel	1 (4.3)	0 (0.0)	0.307
Warfarin	2 (8.7)	3 (5.8)	0.489
Other anticoagulant	1 (4.3)	0 (0.0)	0.307
Liver cirrhosis, n (%)	1 (4.3)	1 (1.9)	0.522
Bystander CPR, n (%)	13/22 (59.1)	25/49 (51.0)	0.528
Dr.Car activation, n (%)	5 (21.7)	11 (21.2)	0.589
Initial EMS rhythm, n (%)			0.068
VF/VT	1 (4.3)	2/50 (4.0)	
PEA	8 (34.8)	6/50 (12.0)	
Asystole	14 (60.9)	42/50 (84.0)	
Initial ED rhythm, n (%)			0.686
VF/VT	2 (8.7)	2 (3.8)	
PEA	4 (17.4)	9 (17.3)	
Asystole	17 (73.9)	41 (78.8)	
From onset to blood sampling in ED, n (%)			0.111
≤30 mins	5 (21.7)	6 (11.5)	
31–60 mins	10 (43.5)	13 (25.0)	
61–120 mins	1 (4.3)	15 (28.8)	
≥121 mins	6 (26.1)	16 (30.8)	
Unknown	1 (4.3)	2 (3.8)	

ROSC: return of spontaneous circulation; IQR: interquartile range; CPR: cardiopulmonary resuscitation; EMS: emergency medical service; VF: ventricular fibrillation; VT: ventricular tachycardia; PEA: pulseless electrical activity; ED: emergency department; mins: minutes.

All continuous variables are represented as median (IQR; Q1-Q3), and categorical variables as number (percentages). Values of P<0.05 are considered to be significant.

### Complete blood counts, standard coagulation tests and lactate level

The results of standard laboratory tests are shown in Table 2. Median platelet count of the ROSC group was significantly higher than that of the non-ROSC group (13.3x10^4^/μL vs. 10.2x10^4^/μL; p = 0.034). Median APTT of ROSC group was significantly shorter than that of the non-ROSC group (59.6 s vs. 75.4 s; p = 0.014). The ROSC group showed higher fibrinogen level (279 mg/dL vs.208 mg/dL; p = 0.004) and lower FDP (52.7 μg/mL vs. 345.8 μg/mL; p = 0.001) and DD (18.42 μg/mL vs. 105.70 μg/mL; p = 0.001) compared with the non-ROSC group. On the other hand, WBC, Hb and PT-INR were not significantly different between the two groups.

**Table 2:** Standard blood tests and ROTEM findings between the ROSC and non-ROSC groups.

Variables	ROSC n = 23	non-ROSC n = 52	p
WBC, /μL, median (IQR)	9000 (6100–12200)	9150 (7025–10450)	0.818
Hb, g/dL, median (IQR)	10.7 (8.7–12.5)	11.6 (9.6–13.7)	0.066
Platelet, 10^4^/μL, median (IQR)	13.3 (10.5–18.5)	10.2 (6.6–14.6)	0.034
PT-INR, median (IQR)	1.61 (1.22–2.12)	1.69 (1.31–2.13)	0.524
APTT, second, median (IQR)	59.6 (45.1–72.7)	75.4 (53.4–132.7)	0.014
Fibrinogen, mg/dL, median (IQR)	279 (241–403)	208 (100–288)	0.004
FDP, μg/mL, median (IQR)	52.7 (11.1–104.0)	345.8 (53.5–1641.3)	0.001
DD, μg/mL, median (IQR)	18.42 (5.44–42.59)	105.70 (25.73–668.38)	0.001
Lactate, mmol/L, median (IQR)	11.3 (9.4–17.0)	16.0 (13.1–19.3)	0.017
EXTEM			
CT, second, median (IQR)	75 (61–107)	97 (67–1685)	0.032
CFT, second, median (IQR)	94 (62–165)	127 (92–206)	0.081
alpha angle, °, median (IQR)	74 (61–78)	67 (55–72)	0.048
A10, mm, median (IQR)	49 (42–60)	42 (22–52)	0.021
A20, mm, median (IQR)	56 (47–65)	47 (15–58)	0.032
A30, mm, median (IQR)	57 (42–67)	41 (13–53)	0.005
MCF, mm, median (IQR)	58 (50–67)	48 (34–59)	0.009
LI30, %, median (IQR)	100 (96–100)	99 (27–100)	0.092
ML, %, median (IQR)	98 (8–100)	100 (27–100)	0.118
FIBTEM			
CT, second, median (IQR)	77 (56–112)	106 (66–5118)	0.037
Hyperfibrinolysis by ROTEM, n (%)	18 (78.3)	49 (94.2)	0.053

ROTEM: rotational thromboelastometry; ROSC: return of spontaneous circulation; WBC: white blood cell; IQR: interquartile range; Hb: hemoglobin; PT-INR: prothrombin time-international normalized ratio; APTT: activated partial thromboplastin time; FDP: fibrin degradation products; DD: D-dimer; CT: clotting time; CFT: clot formation time; A: amplitude; MCF: maximum clot firmness; LI: lysis index; ML: maximum lysis.

All continuous variables are represented as median (IQR; Q1-Q3), and categorical variables as number (percentages). Values of P<0.05 are considered to be significant.

### The results of ROTEM findings

Table 2 also shows the results of the ROTEM test. The ROSC group indicated significantly shorter median CT of EXTEM than the non-ROSC group (75 s vs. 97 s; p = 0.032). The median alpha angle of the ROSC group was significantly higher than that of the non-ROSC group (74° vs. 67°; p = 0.048). The parameters on clot firmness (A10, A20, A30 and MCF) were significantly higher in the ROSC group compared with the non-ROSC group (49 mm vs. 42 mm; p = 0.021, 56 mm vs. 47 mm; 0.032, 57 mm vs. 41 mm; 0.005 and 58 mm vs. 48 mm; p = 0.009, respectively). Other parameters including CFT, LI30, and ML did not reveal any statistical differences. In addition to EXTEM, the ROSC group also indicated significantly shorter CT of FIBTEM than the non-ROSC group (77 s vs. 106 s; p = 0.037). We did not detect a statistical difference in the presence of hyperfibrinolysis between the two groups (78.3% vs. 94.2%; p = 0.053), which was diagnosed by ROTEM.

### Multivariate analysis and calculating the cut-off values by ROC analysis

**Fig 2:**
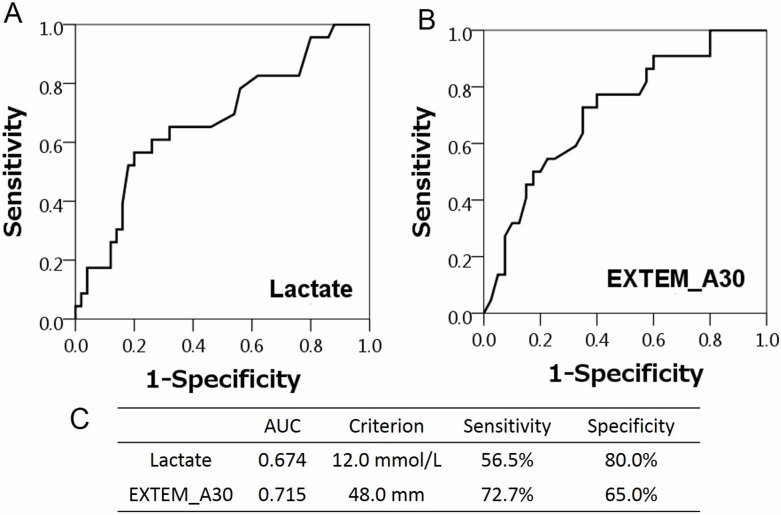
Receiver-Operating Characteristic (ROC) curve analysis of lactate (A and C) and EXTEM A30 (B and C) for Return of Spontaneous Circulation (ROSC) in the emergency department. The cut-off value of lactate was 12.0 mmol/L, the area under the curve (AUC) was 0.674, sensitivity was 56.5%, and specificity was 80.0%. The cut-off value of A30 of EXTEM was 48.0 mm, the AUC was 0.715, sensitivity was 72.7%, and specificity was 65.0%.

We performed multivariate analysis using statistically significant parameters by univariate analysis between the ROSC and non-ROSC groups. Logistic regression analysis demonstrated that the lactate value [odds ratio (OR) 0.880, 95% confidence interval (CI) 0.785–0.986, p = 0.028] and the A30 of the EXTEM test [OR 1.039, 95% CI 1.010–1.070, p = 0.009] were independent predictors for ROSC in the ED (Table 3). Next, we calculated the cut-off values of lactate and CT of EXTEM by the receiver-operating characteristic (ROC) curve analysis of ROSC. The cut-off value of lactate was 12.0 mmol/L, the area under the curve (AUC) was 0.674, sensitivity was 56.5%, and specificity was 80.0% (Fig 2A and 2C). In addition, the cut-off value of A30 of EXTEM was 48.0 mm, the AUC was 0.715, sensitivity was 72.7%, and specificity was 65.0% (Fig 2B and 2C).

**Table 3:** Logistic regression analysis for ROSC in the ED.

	Partial regression coefficient	p value	Odds ratio	95%CI
Lactate	-0.128	0.028	0.880	(0.785–0.986)
EXTEM_A30	0.039	0.009	1.039	(1.010–1.070)
Constant	-0.393	0.700	0.675	

ROSC: return of spontaneous circulation; ED: emergency department; CI: confidence interval.

Values of P<0.05 are considered to be significant.

### The ROTEM-based predictors of ROSC in ED

**Fig 3:**
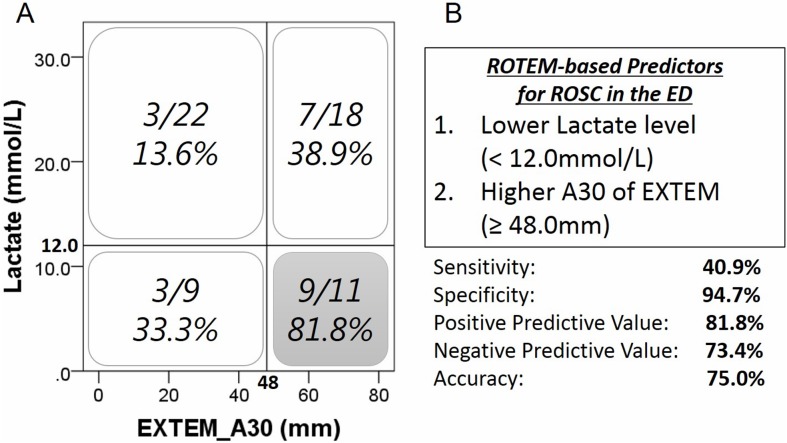
**Relationship between ROSC ratio and independent predictors by multivariate analysis (A). The “novel” ROTEM-based predictors for ROSC in the emergency department and its accuracy (B).** ROTEM, rotational thromboelastometry; ROSC, return of spontaneous circulation; ED, emergency department.

We evaluated both parameters in all cases to determine which values were reliable predictors for ROSC in OHCA patients (Fig 3). The population with low lactate value (<12 mmol/L) and high A30 (> = 48 mm) had the highest successful rate for ROSC (81.8%). On the other hand, high lactate value (> = 12 mmol/L) and low A30 (<48 mm) had the lowest successful rate for ROSC (13.6%) and the worst clinical outcome.

Next, we created ROTEM-based predictors of ROSC in the ED utilizing these cut-off values of significant parameters by multivariate analysis. We defined the positive criteria to be met for the blood samples of OHCA patients as follows: lower lactate level (<12.0 mmol/L) and higher A30 of EXTEM (≥48.0 mm) (Fig 3). These novel criteria for ROSC show low sensitivity (40.9%), high specificity (94.7%) and accuracy (75.0%).

## Discussion

We conducted a retrospective single-center observational study measuring patients’ characteristics, complete blood counts, and standard coagulation tests, as well as viscoelastic tests that reflect the extrinsic coagulation cascade. These findings demonstrated that the lactate level and clot firmness of 30 minutes after initiation of clot formation by EXTEM test were independent predictor for ROSC in patients with OHCA. The ROTEM-based predictors we created had high specificity and accuracy in the acute phase of cardiopulmonary resuscitation. To the best of our knowledge, no previous reports ever focused on ROSC and coagulopathy using whole-blood viscoelastic testing such as ROTEM. These results will help medical personnel find the requirement for advanced post resuscitative treatments such as therapeutic hypothermia for patients by referring to two simple parameters within approximately 30 minutes after admission to the ED. Out of 23 ROSC patients after ED admission, 12 patients (52.2%) were survived for more than 6 hours after ROSC, 6 patients (26.1%) were for more than 24 hours, and only 1 patient (4.3%) with cerebral performance categories (CPC) 1 was discharged from our hospital (data not shown). In addition, our results may support to the decision-making for termination of futile cardiopulmonary resuscitation, which may be beneficial for the allocation of limited healthcare resources.

OHCA has numerous causes, which makes it difficult for emergency physicians and paramedics to understand its pathophysiology. Myocardial infarction and pulmonary embolism are the most common etiologies for adult OHCA patients, which accounted for over 70% [1, 6, 11]. Previous reports using thrombolytic agents such as tissue plasminogen activator (t-PA) showed thrombolysis therapy during cardiac resuscitation was related to higher ROSC ratio compared with control [8, 10, 12]. However, the randomized control trial on the effectiveness of t-PA for 233 cardiac arrest patients with pulseless electric activities demonstrated fibrinolysis therapy did not improve the percentage of ROSC or clinical outcomes [5]. A meta-analysis in 2006 concluded that thrombolytic therapy was related to 2.5 times as many ROSC cases compared to control, although there were significantly more cases of severe bleeding in the thrombolytic group [7]. Unfortunately, the TROICA study in 2008 did not show any advantage of thrombolytic agents for better clinical outcome, including ROSC in OHCA cases with possible cardiac origins. On the contrary, it only indicated that this therapy was correlated with a significantly higher rate of intracranial hemorrhage [6]. There have not been any large-scale studies conducted after the TROICA study.

The present study indicated that both lactate level and a parameter of ROTEM (A30 in EXTEM test) are independent predictors of ROSC, and their combination is strongly connected with the possibility of ROSC. Both parameters, known as point-of-care testing, are measured in the ED in our hospital. The lactate level can be measured within 5 minutes. It only takes 1 minute for the ROTEM to start measurement of EXTEM, 1 to 2 minutes for CT of EXTEM, and 30 minutes for the A30. Therefore, the result of A30 should be ready in less than 35 minutes, suggesting that all important parameters are accessible in a matter of half an hour during cardiac resuscitation in the ED. These findings enable us to promptly determine the indication of aggressive treatment for post cardiac arrest syndrome (PCAS).

The present study has some drawbacks. Firstly, this is retrospective study of one single center with a relatively small sample size. In the emergency department setting, we simply do not have medical personnel that operate ROTEM. In fact, ROTEM analysis was missing for 76% of the patients because only a few physicians (not all physicians) could handle the ROTEM, and physicians on shift had no time for operating the ROTEM during CPR. In addition, there are some missing data about prehospital information and laboratory testing. Vasopressors including adrenalin and vasopressin affect the constriction of systemic blood vessels. Unfortunately, we couldn’t analyze the doses of the vasopressors. Finally, there is no institutional guideline for discontinuation of aggressive cardiopulmonary resuscitation. In our hospital, every physician on call decides on the resuscitative management for the OHCA patient.

Is an anti-thrombolytic therapy the best for the OHCA patients? To date, thrombolytic therapy, including t-PA, has some evidence for the improvement of the clinical outcome for myocardial infarction and pulmonary embolism [7, 8, 10, 12]. However, recent article using ROTEM in 2013 documented that hyperfibrinolysis is observed in many more patients (35.8%) compared with previous reports [19]. Surprisingly, the current study estimated that 89.3% of OHCA patients showed hyperfibrinolysis on admission although no statistical difference was found between each group. ROTEM can detect multiple aspects of thrombogenicity and thrombolysis in real time. The viscoelastic testing using whole blood shows potential for a reliable predictor of coagulopathy in patients with OHCA.

## Conclusions

The new diagnostic criteria including lactate level and the ROTEM parameter of clot firmness predicts a possibility of ROSC in the ED for adult patients with OHCA. In the future, thromboelastometric analysis using larger sample populations will be warranted in order to evaluate the relationships between OHCA induced coagulopathy, including hyperfibrinolysis, and short-term clinical outcome.

## References

[1] Silfvast T. Cause of death in unsuccessful prehospital resuscitation. Journal of internal medicine. 1991;229(4):331–5. 2026986 10.1111/j.1365-2796.1991.tb00355.x

[2] Bottiger BW, Motsch J, Bohrer H, Boker T, Aulmann M, Nawroth PP, et al Activation of blood coagulation after cardiac arrest is not balanced adequately by activation of endogenous fibrinolysis. Circulation. 1995;92(9):2572–8. 7586359 10.1161/01.cir.92.9.2572

[3] Gando S, Nanzaki S, Morimoto Y, Kobayashi S, Kemmotsu O. Tissue factor and tissue factor pathway inhibitor levels during and after cardiopulmonary resuscitation. Thrombosis research. 1999;96(2):107–13. 10574588 10.1016/s0049-3848(99)00073-0

[4] Viersen VA, Greuters S, Korfage AR, Van der Rijst C, Van Bochove V, Nanayakkara PW, et al Hyperfibrinolysis in out of hospital cardiac arrest is associated with markers of hypoperfusion. Resuscitation. 2012;83(12):1451–5. 10.1016/j.resuscitation.2012.05.008 22634432

[5] Abu-Laban RB, Christenson JM, Innes GD, van Beek CA, Wanger KP, McKnight RD, et al Tissue plasminogen activator in cardiac arrest with pulseless electrical activity. The New England journal of medicine. 2002;346(20):1522–8. 10.1056/NEJMoa012885 12015391

[6] Bottiger BW, Arntz HR, Chamberlain DA, Bluhmki E, Belmans A, Danays T, et al Thrombolysis during resuscitation for out-of-hospital cardiac arrest. The New England journal of medicine. 2008;359(25):2651–62. 10.1056/NEJMoa070570 19092151

[7] Li X, Fu QL, Jing XL, Li YJ, Zhan H, Ma ZF, et al A meta-analysis of cardiopulmonary resuscitation with and without the administration of thrombolytic agents. Resuscitation. 2006;70(1):31–6. 10.1016/j.resuscitation.2005.11.016 16762481

[8] Lederer W, Lichtenberger C, Pechlaner C, Kroesen G, Baubin M. Recombinant tissue plasminogen activator during cardiopulmonary resuscitation in 108 patients with out-of-hospital cardiac arrest. Resuscitation. 2001;50(1):71–6. 11719132 10.1016/s0300-9572(01)00317-3

[9] Janata K, Holzer M, Kurkciyan I, Losert H, Riedmuller E, Pikula B, et al Major bleeding complications in cardiopulmonary resuscitation: the place of thrombolytic therapy in cardiac arrest due to massive pulmonary embolism. Resuscitation. 2003;57(1):49–55. 12668299 10.1016/s0300-9572(02)00430-6

[10] Kurkciyan I, Meron G, Sterz F, Janata K, Domanovits H, Holzer M, et al Pulmonary embolism as a cause of cardiac arrest: presentation and outcome. Archives of internal medicine. 2000;160(10):1529–35. 10826469 10.1001/archinte.160.10.1529

[11] Keuper W, Dieker HJ, Brouwer MA, Verheugt FW. Reperfusion therapy in out-of-hospital cardiac arrest: current insights. Resuscitation. 2007;73(2):189–201. 10.1016/j.resuscitation.2006.08.030 17239516

[12] Bozeman WP, Kleiner DM, Ferguson KL. Empiric tenecteplase is associated with increased return of spontaneous circulation and short term survival in cardiac arrest patients unresponsive to standard interventions. Resuscitation. 2006;69(3):399–406. 10.1016/j.resuscitation.2005.09.027 16563599

[13] Nakayama Y, Nakajima Y, Tanaka KA, Sessler DI, Maeda S, Iida J, et al Thromboelastometry-guided intraoperative haemostatic management reduces bleeding and red cell transfusion after paediatric cardiac surgery. British journal of anaesthesia. 2015;114(1):91–102. 10.1093/bja/aeu339 25303988

[14] Alamo JM, Leon A, Mellado P, Bernal C, Marin LM, Cepeda C, et al Is "Intra-operating Room" Thromboelastometry Useful in Liver Transplantation? A Case-Control Study in 303 Patients. Transplantation proceedings. 2013;45(10):3637–9. 10.1016/j.transproceed.2013.11.008 24314981

[15] Schochl H, Nienaber U, Hofer G, Voelckel W, Jambor C, Scharbert G, et al Goal-directed coagulation management of major trauma patients using thromboelastometry (ROTEM)-guided administration of fibrinogen concentrate and prothrombin complex concentrate. Critical care. 2010;14(2):R55 10.1186/cc8948 20374650 PMC2887173

[16] Koami H, Sakamoto Y, Sakurai R, Ohta M, Imahase H, Yahata M, et al The thromboelastometric discrepancy between septic and trauma induced disseminated intravascular coagulation diagnosed by the scoring system from the Japanese association for acute medicine. Medicine (Baltimore). 2016;95(31):e4514. 27495106 10.1097/MD.0000000000004514 PMC4979860

[17] Davenport R, Manson J, De'Ath H, Platton S, Coates A, Allard S, et al Functional definition and characterization of acute traumatic coagulopathy. Critical care medicine. 2011;39(12):2652–8. 10.1097/CCM.0b013e3182281af5 21765358 PMC3223409

[18] Olde Engberink RH, Kuiper GJ, Wetzels RJ, Nelemans PJ, Lance MD, Beckers EA, et al Rapid and correct prediction of thrombocytopenia and hypofibrinogenemia with rotational thromboelastometry in cardiac surgery. Journal of cardiothoracic and vascular anesthesia. 2014;28(2):210–6. 10.1053/j.jvca.2013.12.004 24630470

[19] Schochl H, Cadamuro J, Seidl S, Franz A, Solomon C, Schlimp CJ, et al Hyperfibrinolysis is common in out-of-hospital cardiac arrest: results from a prospective observational thromboelastometry study. Resuscitation. 2013;84(4):454–9. 10.1016/j.resuscitation.2012.08.318 22922072

[20] Ahn Y, Goerlinger K. Coagulopathy and Hypercoagulability In: Wiener-Kronish JP, senior editor. Critical Care Handbook of the Massachusetts General Hospital. 6th ed Philadelphia: Wolters Kluwer; 2016 pp. 322–350.

